# A randomised phase II study of pegylated arginine deiminase (ADI-PEG 20) in Asian advanced hepatocellular carcinoma patients

**DOI:** 10.1038/sj.bjc.6605856

**Published:** 2010-08-31

**Authors:** T-S Yang, S-N Lu, Y Chao, I-S Sheen, C-C Lin, T-E Wang, S-C Chen, J-H Wang, L-Y Liao, J A Thomson, J Wang-Peng, P-J Chen, L-T Chen

**Affiliations:** 1 Department of Internal Medicine, Chang Gung Memorial Hospital, LinKou Medical Center, Chang Gung University, Taoyuan 33305, Taiwan; 2 Department of Internal Medicine, Kaohsiung Chang Gung Memorial Hospital, Kaohsiung 833, Taiwan; 3 Cancer Center, Taipei Veterans General Hospital, Taipei 112, Taiwan; 4 Department of Internal Medicine, Mackay Memorial Hospital, Taipei 10449, Taiwan; 5 Department of Internal Medicine, Kaohsiung Medical University Hospital, Kaohsiung Medical University, Kaohsiung 807, Taiwan; 6 Department of Internal Medicine, Ren-Ai Branch, Taipei City Hospital, Taipei 106, Taiwan; 7 Polaris Pharmaceuticals Inc., San Diego, CA, USA; 8 National Institute of Cancer Research, National Health Research Institutes, 2F, No. 367, Sheng-Li Road, Tainan 704, Taiwan; 9 Department of Internal Medicine, National Taiwan University Hospital, Taipei 100, Taiwan; 10 Department of Internal Medicine, National Cheng Kung University Hospital and College of Medicine, Tainan 704, Taiwan

**Keywords:** arginine, arginine deiminase, hepatocellular carcinoma, polyethylene glycol, argininosuccinate synthetase

## Abstract

**Background:**

Human hepatocellular carcinoma (HCC) cells are largely deficient of argininosuccinate synthetase and thus auxotrophic for arginine. This study aims to investigate the efficacy and pharmacodynamics of pegylated arginine deiminase (ADI-PEG 20), a systemic arginine deprivation agent, in Asian HCC patients.

**Methods:**

Patients with advanced HCC who were not candidates for local therapy were eligible and randomly assigned to receive weekly intramuscular injections of ADI-PEG 20 at doses of 160 or 320 IU m^−2^. The primary end point was disease-control rate (DCR).

**Results:**

Of the 71 accruals, 43.6% had failed previous systemic treatment. There were no objective responders. The DCR and the median overall survival (OS) of the intent-to-treat population were 31.0% (95% confidence interval (CI): 20.5–43.1) and 7.3 (95% CI: 4.7–9.9) months respectively. Both efficacy parameters were comparable between the two study arms. The median OS of patients with undetectable circulating arginine for more than or equal to and <4 weeks was 10.0 (95% CI: 2.1–17.9) and 5.8 (95% CI: 1.4–10.1) months respectively (*P*=0.251, log-rank test). The major treatment-related adverse events were grades 1–2 local and/or allergic reactions.

**Conclusions:**

ADI-PEG 20 is safe and efficacious in stabilising the progression of heavily pretreated advanced HCC in an Asian population, and deserves further exploration.

Hepatocellular carcinoma (HCC) is one of the most common malignancies in the world (Ryder, 2003; Sun *et al*, 2003; Yu and Keeffe, 2003; Llovet, 2005; El-Serag and Rudolph, 2007). There are approximately 500 000 new cases diagnosed annually worldwide. The majority of them are diagnosed with advanced disease that is not amenable to effective local therapies such as liver transplantation, resection, percutaneous ablation or transcatheter arterial chemoembolisation (TACE) therapy. Systemic chemotherapy, for instance the conventional ‘standard’ therapy of doxorubicin, has been largely ineffective and typically associated with many side effects (Di Maio *et al*, 2002; Ryder, 2003; Llovet, 2005; Chen *et al*, 2006). As a result, placebo has remained as an acceptable control in randomised phase III trials until sorafenib (Nexavar; Bayer HealthCare AG, Leverkusen, Germany) was shown to improve the survival of advanced HCC over placebo in both Caucasian and Asian populations (Llovet *et al*, 2008; Cheng *et al*, 2009). Despite odds ratio of 0.6–0.7 compared with placebo-control in both studies, the absolute improvement was not more than 2–3 months. Therefore, pursuing additional options of effective and tolerable systemic therapies for advanced HCC is still mandatory (O'Neill and Venook, 2007; Abou-Alfa and Venook, 2008; Zhu, 2008).

Arginine is a non-essential amino acid for humans and mice (Rogers, 1994; Tapiero *et al*, 2002). It is synthesised in humans and other mammals from citrulline in two steps through the urea cycle enzymes, argininosuccinate synthetase (ASS) and argininosuccinate lyase (ASL). ASS catalyses the conversion of citrulline and aspartic acid to argininosuccinate, which is then converted to arginine and fumaric acid by ASL. It has long been known that some tumour cells are auxotrophic for arginine, based on the observations that normal cells derived from liver, kidney and testes could grow in medium depleted of arginine but supplemented with citrulline, whereas tumour cells from these organs could not (Tytell and Neuman, 1960). Other investigators reported that certain tumour cell lines could not be maintained in medium contaminated with *Mycoplasma* species, which could be attributed to arginine depletion mediated by *Mycoplasma*-produced arginine deiminase (ADI; Kenny and Pollock, 1963; Kraemer *et al*, 1963; Kraemer, 1964; Sugimura *et al*, 1990). These observations indicated that ADI, through depletion of circulating arginine, might be a potential anti-cancer agent. The enzyme was cloned and expressed in *E. coli* and subsequently conjugated to polyethylene glycol (PEG) to increase the circulating half-life and decrease the immunogenicity of the recombinant Mycoplasma enzyme (Takaku *et al*, 1992, 1993; Holtsberg *et al*, 2002).

It has been shown that some human HCC and melanoma cell lines and tissue samples do not express ASS, making them auxotrophic for arginine and thus reasonable candidates for arginine deprivation therapy (Ensor *et al*, 2002; Dillon *et al*, 2004). The first phase I/II study of pegylated arginine deiminase (ADI-PEG 20) for unresectable HCC was reported in 2004 (Izzo *et al*, 2004). In that dose-escalating study, 160 IU m^−2^ was selected as the optimal biologic dose for weekly ADI-PEG 20 therapy, as it sustained depletion of circulating arginine for 7 days. Among the 19 enrolled patients, the best tumour response was objective response in 47.4% (two CR, seven PR) and stable disease in 36.8%, and the median survival was 410 days. No significant toxicity was observed even in patients who had persistent arginine depletion for 3 or more months. In a more recent phase II study, Glazer *et al* (2010) showed that this agent could achieve 2.5% of objective response rate and 65% of diseases control rate in 80 Caucasian patients with predominantly hepatitis C-related HCC and minimal prior systemic therapy. This parallel phase II study aims to investigate the therapeutic efficacy of ADI-PEG 20 for advanced HCC in an Asian population.

## Materials and methods

### Trial design and participants

This was an open-label, multi-centre, randomised phase II study undertaken in Taiwan. The primary end point of the study was to assess the therapeutic efficacy of ADI-PEG 20 in an Asian HCC population in terms of disease-control rate (DCR), which was defined as radiographic objective response (complete or partial response) or stable disease for 8 or more weeks after ADI-PEG 20 treatment. The secondary end points were overall survival (OS), progression-free survival (PFS) and pharmacodynamics parameters, that is, changes of circulating level of arginine and citrulline and anti-ADI-PEG 20 antibody. The study was approved by the ethics committee at each trial centre and by the Department of Health, Executive Yuan, Taiwan, and all patients signed written informed consent. The study followed the Declaration of Helsinki Principles and Good Clinical Practice guidelines.

### Eligibility

Patients with measurable, metastatic or locally advanced HCC that had failed or were too advanced to have definitive local therapy, that is, surgical resection, percutaneous ethanol or acetic acid injection, TACE, radiofrequency ablation either alone or in combination, were eligible. Patients were considered to have unresectable HCC if their tumours were of either local therapy-failed Barcelona Criteria for Liver Cancer stage B or BLCL stage C (the presence of either major vascular invasion or distant metastases, or in combination). The diagnosis of HCC had to have been established by cytology or histopathology. Patients were also required to be age of ⩾18 years, and have an expected survival of ⩾12 weeks, Karnofsky performance score ⩾80, Child–Pugh score ⩽8, serum levels of total bilirubin <2.0 mg per 100 ml, albumin >2.5 g per 100 ml, AST/ALT and alkaline phosphatase <5 × upper limit of normal (ULN), ammonia <70 *μ*g per 100 ml, glucose >60 mg per 100 ml, amylase <1.5 × ULN, uric acid ⩽8 mg per 100 ml (with or without medication control), peripheral blood absolute neutrophil count ⩾1500 per *μ*l and platelets ⩾50 000 per *μ*l. Female patients of childbearing age and male patients had to use appropriate contraception during the study period. A serum HCG pregnancy test had to be negative before entry. Previous chemotherapy and/or targeted therapy were allowed, but had to be 4 weeks or more (6 weeks for nitrosoureas or mitomycin C) before entering the study, and patients were to be completely recovered from adverse events. Other exclusion criteria included the presence of uncontrolled inter-current illness and a history of other malignancies within 5 years of study entry.

### Therapy and clinical assessments

Enrolled patients would be randomly assigned to receive intramuscular injection of ADI-PEG 20 (Polaris Pharmaceuticals Inc., San Diego, CA, USA) at 160 or 320 IU m^−2^ on days 1, 8, 15 and 22 every 4 weeks (as one cycle). Medical history and physical examination, serum *α*-fetoprotein and uric acid levels, and circulating arginine and citrulline levels were assessed before each ADI-PEG 20 administration. Complete blood count, blood biochemistry tests, coagulation test, urinary analyses and anti-ADI-PEG 20 antibody titre were assessed after every cycle (4 weeks) of treatment. Circulating arginine and citrulline levels were determined by mass spectrometry assays, and the plasma anti-ADI-PEG 20 antibody level was assessed by the ELISA-based immunogenicity assay. These pharmacodynamic parameter determinations were performed in outsourced central laboratories. Grading of adverse events and assessment of tumour response were performed based on the National Cancer Institute Common Toxicity Criteria, Version 3.0 and the RECIST criteria respectively (Therasse *et al*, 2002). All the treatments were given until a maximum six cycles (24 weeks) of treatment were reached unless unacceptable toxicity, the refusal of patient, withdrawal of informed consent or death was observed. Those who remained progression free after six cycles of treatment could continue the therapy in an expanded, compassionate use programme.

### Statistical considerations

The primary end point of the study was DCR, which was defined as the ratio of patients with objective tumour response (complete or partial response) or stable disease for ⩾8 weeks in either dosing arm. Secondary end points were OS, PFS and changes in pharmacodynamic parameters. According to Simon's optimal two-stage design for uninterested and interested ORR of 10 and 30%, respectively, with both *α* and *β* errors probabilities of 0.05 and 0.20, respectively, 10 patients would be accrued in the first stage of each dosing cohort (Simon, 1989). If <2 responders were observed among the initial 10 evaluable patients in either dosing cohort, then the dosing would be considered as ineffective and the accrual in that cohort would be terminated. If ⩾2 responders were observed among the initial 10 evaluable patients in any dosing cohort, an additional 19 patients would be accrued into the second stage of study. If ⩾6 responses were observed in any dosing cohort, ADI-PEG 20 would be considered effective; otherwise, it would be concluded that ADI-PEG 20 was not effective enough for further exploration in advanced HCC. All patients who received any assigned treatment were included in the intent-to-treat (ITT) population. The definition of an event for OS was death from any cause. The survival distributions were estimated using the Kaplan–Meier method (Kaplan and Meier, 1958). *χ*^2^-Test and Fisher's exact test were used for descriptive variables, and Wilcoxon Mann–Whitney approach was used for continuous variables. Any *P*-values <0.05 (two-sided test) were considered statistically significant.

## Results

### Recruitment and demography of participants

Between October 2006 and March 2008, 71 patients from eight participating hospitals were enrolled into the study; 37 patients were allocated to the 160 IU m^−2^ dosing arm and 34 to the 320 IU m^−2^ dosing arm. There were 59 men (83.1%) and 12 women (16.9%), with a median age of 55 years (range 27–82) and hepatitis B surface antigen seropositive in 49 (69.0%). A total of 63 patients (88.7%) had failed previous therapies, including surgery in 29 (40.8%), one or more sessions of chemoembolisation in 46 (64.8%), previous chemotherapy/targeted therapy in 31 (43.6%) and radiation therapy in 19 (26.8%). Demographics of the patients were well balanced except that significantly more patients in the 320 IU m^−2^ arm had gross vascular invasion in radiography (70.6 *vs* 40.5% in 160 IU m^−2^ arm; *P*=0.017, *χ*^2^-test), as listed in Table 1.

### Toxicity profiles and off-study causes

A total of 205.5 cycles were administered (median, 2.25 cycles; range 0.5–6). At the end of the study, three (8.8%) and five (13.5%) subjects in the 320 and 160 IU m^−2^ arms, respectively, remained in the study and received the maximum of six cycles. The most common treatment-related adverse events were of grade 1–2 and associated with systemic and local allergic reaction and drug-related metabolic changes, that is, hypersensitivity/skin rash in 26.8%, local tissue reaction at injection site in 22.5%, hyperuricemia in 19.7%, pruritus in 15.5%, fatigue in 9.8%, hyperammonemia in 4.2%, fever in 7.0% and diarrhoea in 5.6%, as listed in Table 2. Thirteen patients (18.3%) died during the study or within 1 month after their last treatment. These deaths were attributed to disease progression in 11, hepatic decompensation in 1 and oesophageal varices haemorrhage in another. The clinical characteristics of these 13 patients, at study entry, did not differ from those of the rest of 58 patients who remained alive for more than 1 month after last treatment.

### Pharmacodynamics and immunogenicity

**Figure 1:**
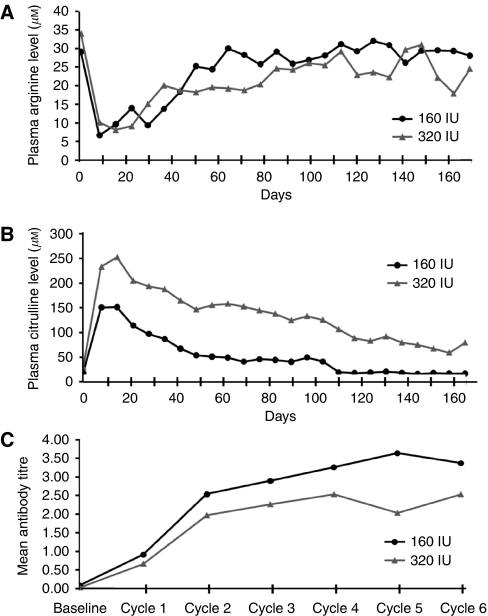
Changes of mean plasma (**A**) arginine and (**B**) citrulline levels, and (**C**) plasma titres of anti-ADI antibody after weekly administration of either 160 or 320 IU m^−2^ ADI-PEG 20 in patients with unresectable HCC.

For both dosing cohorts, the circulating arginine level markedly decreased after the first dose (undetectable in majority of patients on day 8) and then rose gradually with extended time of treatment, which was accompanied by a reciprocal change in circulating citrulline levels, as shown in Figure 1A and B. The circulating arginine level remained at approximately ⩽50% of the baseline level for about 10 weeks and 6 weeks in the 320 and 160 IU m^−2^ cohorts, respectively. Of note, the reported arginine levels were obtained 1 week after the administration of ADI-PEG 20 and thus may not accurately reflect the maximal and the duration of suppression of arginine levels attained during the week.

Antibodies directed against ADI-PEG 20 could be detected before cycle 2 (week 5) and generally reached a plateau before cycle 5 (week 17). The return to baseline levels of circulating arginine closely correlated with the increase in antibody titre (Figure 1C).

### Response and survival

**Figure 2:**
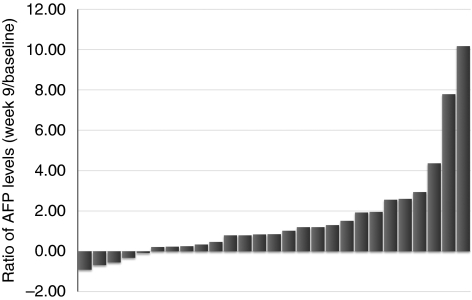
Waterfall plotting of *α*-fetoprotein (AFP) level changes after 8 weeks of ADI-PEG 20 treatment in 27 patients whose baseline AFP above 200 ng ml^−1^.

There were no objective (complete or partial) tumour responders. The best tumour response was stable disease in 22 patients (31.0%), progressive disease in 43 (60.6%) and unevaluable in 6 (8.4%). The DCR (complete/partial response+stable disease) for the ITT populations was 31.0% (22 of 71, 95% confidence interval (CI): 20.5–43.1). Each dose cohort had 11 patients with stable disease as their best response. Among the 22 patients who showed stable disease at the end of cycle 2, six (27.3%) remained progression free at the time of last assessment. As limited objective tumour response is commonly observed in most targeted agent trials, we also assessed the therapeutic efficacy of ADI-PEG 20 by monitoring changes of the serum AFP levels. To avoid the influence of non-tumour-originating AFP fluctuations (ie, those associated with hepatitis activity), we only assessed AFP response in patients with baseline AFP levels above 200 ng ml^−1^ (Liaw *et al*, 1986; Chen *et al*, 2009) and received treatments for at least 8 weeks. Of these 27 patients, the changes in AFP level after 8 weeks of ADI-PEG 20 treatment were ⩾50% reduction in 3 (11.1%), <50% reduction in 2 (4.7%), less than onefold increase in 9 (33.3%), one- to twofold increase in 7 (25.9%) and more than twofold increase in 6 (22.2%), as shown in Figure 2.

**Figure 3:**
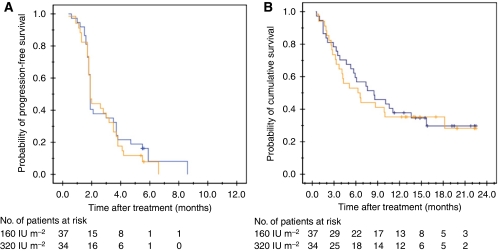
Progression-free (**A**) and overall survival (**B**) curves according to the ADI-PEG 20 dose administered, 160 IU m^−2^ (blue line) and 320 IU m^−2^ (orange line), with *P*-values of 0.73 and 0.76 (log-rank test), respectively.

The median duration of disease control was 2.8 months for both dose cohorts, with the 95% CI of 1.4–4.3 months in 160 IU m^−2^ cohort and 2.4–3.3 months in 320 IU m^−2^ cohort. The median PFS and OS of the ITT population were 1.8 (95% CI: 1.8–3.0) months and 7.3 (95% CI: 4.7–9.9) months respectively. There was no statistical difference for both PFS and OS between the two study cohorts, with *P*-values 0.73 and 0.76 (log-rank test) respectively, as listed in Table 3. The survival curves of the two dose cohorts are plotted in Figure 3A and B.

The median survival of patients with baseline AFP level of <10^2^ (*N*=26), 10^2^ to <10^3^ (*N*=12), 10^3^ to <10^4^ (*N*=14) and >10^4^ (*N*=19) ng ml^−1^ was 11.8 (95% CI: 3.3–20.3) months, 15.7 (95% CI: 7.6–23.8) months, 4.3 (95% CI: 0–8.8) months and 4.1 (95% CI: 3.0–5.2) months, respectively (*P*=0.0006, log-rank test).

**Figure 4:**
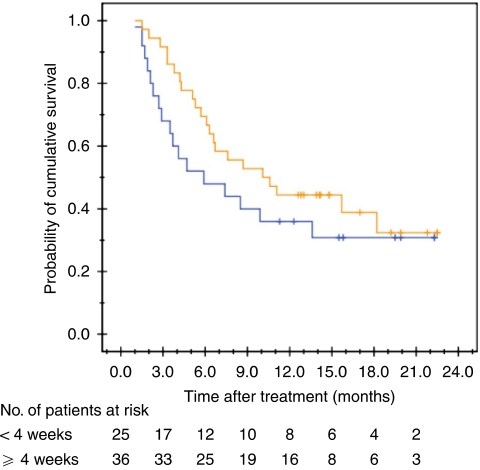
Overall survival curves in patients with depletion of circulating arginine for ⩾4 weeks (orange line) and <4 weeks (blue line) of duration after ADI-PEG 20 treatment (*P*=0.251, log-rank test).

The proposed mechanism of action of ADI-PEG 20 treatment is the preferential starvation of tumour cells by arginine depletion, so the correlation between the duration of arginine depletion and OS was examined. Among the 61 subjects who had received at least one complete cycle (four doses) of treatment, the median survival of those with (*n*=36) and without (*n*=25) sustained depletion of plasma arginine level for 4 weeks was 10.0 (95% CI: 2.1–17.9) months and 5.8 (95% CI: 1.4–10.1) months respectively (*P*=0.251, log-rank test; Figure 4). Of the 36 patients showing depletion of plasma arginine for 4 weeks, 13 patients continued to show depletion of plasma arginine for 8 or more weeks. The median OS of these 13 patients was 15.2 months (95% CI: 1.2–29.1).

In addition, we also explored the correlation between the expression of ASS in tumour tissue and OS. Formalin-fixed, paraffin-embedded archival tumour specimens from 44 patients were subjected to immunohistochemical study using anti-ASS monoclonal antibody (Polaris Pharmaceuticals Inc.) and visualised by a biotinylated horse anti-mouse, streptavidin–HRP system (Vector Labs Inc., Burlingame, CA, USA). Of them, 33 (75%) were ASS deficient and 11 (25%) were ASS positive. The median OS of all 44 patients was 5.9 (95% CI: 0.6–11.2) months, whereas the median OS of patients with ASS-deficient and ASS-positive tumours was 8.3 (95% CI: 3.4–14.0) and 3.3 (95% CI: 1.3–2.5) months (*P*=0.065, log-rank test).

## Discussion

In this study, we have shown that weekly intramuscular injection of ADI-PEG 20 at doses of 160 or 320 IU m^−2^ could achieve a DCR of 31.0% (95% CI: 20.5–43.1) and an OS of 7.3 (95% CI: 4.7–9.9) months in a previously heavily treated Asian advanced HCC population. The majority of the patients enrolled in this study (88.7%) had failed one or more previous treatment modalities for HCC, including systemic therapy in 43.6% patients. As compared with the initial reports in smaller Caucasian cohorts, minimal objective tumour response was observed in the studies of Glazer *et al* (2010) and ours (Curley *et al*, 2003; Izzo *et al*, 2004). However, the DCRs in current study and the Caucasian phase II study were 31 and 63% respectively (Glazer *et al*, 2010). The causes for discrepancy remain unknown, but may be attributed to the higher percentage of patients with previous systemic treatment in our study (43.6 *vs* 1.3%) and/or a potential difference in how HBV- and HCV-related HCC respond to the arginine deprivation therapy. Unfortunately, Glazer *et al* reported their survival data as mean survival after the diagnosis of unresectable HCC, which makes the comparison of survival after treatment between the two studies impossible.

However, the DCR and OS observed in our study were comparable with that of 35% and 6.5 (95% CI: 5.6–7.6) months for patients who received sorafenib in a phase III study in Asia-Pacific region (Cheng *et al*, 2009). In comparison, 43.6% of our patients had failed previous systemic therapy including chemotherapy and anti-angiogenic therapy, which was not allowed in the Asia-Pacific sorafenib trials. In addition, ADI-PEG 20 was relatively well tolerated as compared with sorafenib. The major toxicities of ADI-PEG 20 were of grades 1–2 local and/or allergic reactions, which were distinct from those adverse events observed in association with cytotoxic or molecular targeted agents. However, the impact of ADI-PEG 20 on the natural course of advanced HCC has to be validated further by randomised phase III trials. Furthermore, the effectiveness and non-overlapping toxicity of ADI-PEG 20 make it a potential candidate to be combined with other promising agent(s) for future clinical trials in advanced HCC.

Our pharmacodynamic analysis confirmed that weekly treatment with 160 IU m^−2^ was sufficient to deplete circulating arginine for 7 days (Izzo *et al*, 2004). The 320 IU m^−2^ dose in this study was chosen to investigate if a further increase in dosage would result in an even more prolonged depletion of arginine and thus possibly greater efficacy. Anti-ADI-PEG 20 antibody was detectable after week 4 in some patients, and its frequency and titre increased after repetitive administration of ADI-PEG 20, which was accompanied by a gradual return of the circulating arginine levels in both study arms. The median period of sustained reduction of circulating arginine to <50% of baseline levels in the 160 and 320 IU m^−2^ dose groups was approximately 6 weeks and 10 weeks respectively. However, the DCR and OS were similar in both dose cohorts. It should be noted that the circulating arginine levels reported in this study were obtained 7 days after administration of the study drug. The extent to which arginine was depleted immediately after administration of the study drug and the duration of this level of depletion is not known. It warrants further investigation. However, based on the results of this study, weekly 160 IU m^−2^ should remain as the recommended dosing schedule in future trials.

A *post hoc* analysis of the survival data revealed a potential correlation between duration of arginine deprivation and survival in this limited patient population. Patients with depletion of circulating arginine for ⩾4 weeks had a trend towards better survival than those with depletion of arginine for <4 weeks. This finding is consistent with what has been conceived for anti-angiogenic therapy, an alternative selective starvation therapy (Slaton *et al*, 1999). The return of circulating arginine levels was preceded by the rising titre of anti-ADI-PEG 20 antibody. Therefore, the therapeutic efficacy of ADI-PEG 20 therapy may be further improved by modifying the antigenic epitope of ADI and/or by combining ADI-PEG 20 with immune modulation or cytotoxic therapy to diminish the production of anti-ADI-PEG 20 antibody. Furthermore, there was also a trend towards better survival in patients with ASS-deficient HCC relative to those who were ASS positive. The potential of using ASS status as a predictive biomarker and/or selection criteria for arginine deprivation therapy should be further investigated in a larger patient population study.

Recent reports have reported that lowering peripheral blood arginine can result in anti-angiogenesis by inhibition of nitric oxide synthesis, alteration of immune cell tumour surveillance and induction of autophagy (Gong *et al*, 2003; Bronte and Zanovello, 2005; Shen *et al*, 2006; Peranzoni *et al*, 2007; Bowles *et al*, 2008; Kim *et al*, 2009; Delage *et al*, 2010). These can all be considered as additional mechanisms of action of ADI-PEG 20.

In conclusion, ADI-PEG 20 is safe and efficacious in stabilising the progression of advanced HCC in an Asian population. On the basis of the results of this study, a multi-national phase III study for HCC is being planned. In addition, the exceptional safety profile and the unique mechanisms of action suggest that ADI-PEG 20 is a good candidate to be used in combination with other molecular targeting or cytotoxic agents. The planning for combination studies in patients with other ASS-deficient tumours is currently underway.

## Figures and Tables

**Table 1:** Patient demographics

**Parameter/dose level**	**320 IU m^−2^**	**160 IU m^−2^**	**Overall**
Case, number	34	37	71
			
*Gender*			
Male	30 (88.2%)	29 (78.4%)	59 (83.1%)
Female	4 (11.8%)	8 (21.6%)	12 (16.9%)
			
*Age*			
Median	55	56	55
Range	31–82	27–80	27–82
			
*Karnofsky performance status*			
100	5 (14.7%)	5 (13.5%)	10 (14.1%)
90	21 (61.2%)	28 (75.7%)	49 (69.0%)
80	8 (23.5%)	4 (10.8%)	12 (16.9%)
			
*Child–Pugh class*			
A	31 (91.2%)	34 (91.9%)	65 (91.6%)
B	3 (8.8%)	3 (8.1%)	6 (8.4%)
			
*HbsAg*			
Positive	25 (73.5%)	24 (64.9%)	49 (69.0%)
Negative	9 (26.5%)	13 (35.1%)	22 (31.0%)
			
*Anti-HCV antibody*			
Positive	8 (23.5%)	11 (29.7%)	19 (26.8%)
Negative	24 (76.5%)	25 (67.6%)	51 (71.8%)
Unknown	0 (0.0%)	1 (2.7%)	1 (1.4%)
			
*Vascular invasion*			
Absent	10 (29.4%)	22 (59.5%)	32 (45.1%)
Present	24 (70.6%)	15 (40.5%)	39 (54.9%)
			
*Extrahepatic metastases*			
Absent	15 (44.1%)	15 (40.5%)	30 (42.3%)
Present	19 (55.9%)	22 (59.5%)	41 (57.7%)
			
*Prior therapy*			
Hepatic resection	11 (32.4%)	18 (48.6%)	29 (40.8%)
Transcatheter arterial chemoembolization	21 (61.8%)	25 (67.6%)	46 (64.8%)
Radiotherapy	8 (23.5%)	11 (29.7%)	19 (26.8%)
Systemic therapy	16 (47.0%)	15 (40.5%)	31 (43.6%)
Thalidomide alone	4 (11.8%)	3 (8.1%)	7 (9.9%)
Chemotherapy with/without thalidomide	8 (23.5%)	8 (21.6%)	16 (22.5%)
Anti-angiogenic therapy^a^	4 (11.8%)	4 (10.8%)	8 (11.3%)

Abbreviation: HbsAg=hepatitis B surface antigen.

a Including PI88 in 3, sorafenib in 2, sunitinib in 1 and bevacizumab in 2.

**Table 2:** Treatment-related adverse events

	**320 IU m^−2^ (*N*=34)**		**160 IU m^−2^ (*N*=37)**		**Overall (*N*=71)**	
**Adverse event**	**Grade 1–2**	**Grade 3–4**	**Grade 1–2**	**Grade 3–4**	**Grade 1–2**	**Grade 3–4**
Anaemia (%)	5.9		2.7	2.7	4.2	1.4
Hypersensitivity/rash (%)	20.6		32.4		26.8	
Pruritus (%)	17.6		13.5		15.5	
Local reaction (%)	26.7		18.9		22.5	
Fatigue (%)	5.9	2.9	10.8		8.4	1.4
Fever (%)	8.8		5.4		7.0	
Diarrhea (%)	8.8		2.7		5.6	
Hyperuricemia (%)	11.8	5.9	16.2	5.4	14.1	5.6
Hyperammonemia (%)	5.9		2.7		4.2	

**Table 3:** Therapeutic efficacy

**Items**	**320 IU m^−2^ (*N*=34)**	**160 IU m^−2^ (*N*=37)**	**Overall (*N*=71)**
*Best tumor response,* % *(95*% *CI)*			
Objective response	0	0	0
*Stable disease*			
⩾8 weeks	32.4 (16.6–48.1)	29.7 (15.0–44.4)	31.0 (20.2–41.8)
⩾16 weeks	11.8 (0.9–22.6)	16.2 (0.4–28.1)	14.1 (0.6–22.2)
⩾24 weeks	5.9 (0.0–13.8)	10.8 (0.1–20.8)	8.4 (0.2–14.9)
Median PFS (95% CI), months	1.9 (1.8–3.5)	1.8 (1.8–3.5)	1.8 (1.8–3.0)
Median OS (95% CI), months	6.2 (2.8–9.6)	8.4 (3.6–13.1)	7.3 (4.7–9.9)

Abbreviations: OS=overall survival; PFS=progression-free survival.^[2]^oth OS and PFS were estimated by Kaplan–Meier analyses.
